# Habitual sleep duration, healthy eating, and digestive system cancer mortality

**DOI:** 10.1186/s12916-025-03882-w

**Published:** 2025-01-27

**Authors:** Diana A. Nôga, Elisa M. S. Meth, André P. Pacheco, Jonathan Cedernaes, Pei Xue, Christian Benedict

**Affiliations:** 1 Department of Pharmaceutical Biosciences, Uppsala University, Box 593, Husargatan 3, Uppsala, 751 24 Sweden; 2 Department of Research and Innovation, Division of Mental Health and Addiction, Oslo University Hospital, Sognsvannsveien 21, Oslo, 0372 Norway; 3 Institute of Clinical Medicine, Faculty of Medicine, University of Oslo, Postboks 1039 Blindern, 0315, Oslo, Norway; 4 Department of Medical Sciences, Uppsala University, Akademiska Sjukhuset, Ing. 40, 5 Tr, Uppsala, 751 85 Sweden; 5 Department of Medical Cell Biology, Uppsala University, Box 571, Husargatan 3, Uppsala, 751 24 Sweden

**Keywords:** Lifestyle, Fibre, Fruits, Vegetables, Meat, UK Biobank Cohort, Risk analysis

## Abstract

**Background:**

Lifestyle choices, such as dietary patterns and sleep duration, significantly impact the health of the digestive system and may influence the risk of mortality from digestive system cancer.

**Methods:**

This study aimed to examine the associations between sleep duration, dietary habits, and mortality from digestive system cancers. The analysis included 406,584 participants from the UK Biobank cohort (54.1% women; age range: 38–73 years), with sleep duration classified as short (≤ 6 h, 24.2%), normal (7–8 h, 68.4%), and long (≥ 9 h, 7.4%). Healthy eating habits were defined as a daily intake of at least 25 g of fibre, seven portions of fruits and vegetables, and fewer than four servings of meat per week. These dietary factors were combined into a score ranging from 0 (least healthy) to 3 (healthiest). Cox proportional hazards regression analyses were conducted, with a median follow-up period of 12.6 years, ending on September 30, 2021.

**Results:**

3949 participants died from cancer of the digestive system. Both short and long sleep duration were associated with an increased risk of mortality from cancer of the digestive system (1.09 (1.01–1.18) and 1.14 (1.03–1.27), respectively). Additionally, a diet score ≥ 1 was linked to a lower cancer risk (0.72–0.91 (0.59–0.96)). Adjusting for smoking, type 2 diabetes, and body mass index (BMI) status eliminated the association between sleep duration and digestive cancer mortality. The association between healthy dietary patterns and the risk of digestive system cancer mortality did not vary by sleep duration.

**Conclusions:**

Aberrant sleep durations may increase the risk of mortality from digestive system cancer, potentially through smoking, higher BMI, and type 2 diabetes. However, aberrant sleep durations do not seem to reduce the protective effects of a healthy dietary pattern.

**Supplementary Information:**

The online version contains supplementary material available at 10.1186/s12916-025-03882-w.

## Background

Cancer in the digestive system poses a significant public health burden worldwide, making successful prevention essential for reducing morbidity and mortality [1]. In this context, epidemiological evidence underscores the importance of daily sleep duration [2–6]. For instance, a study from the UK Biobank cohort found that individuals sleeping more than 9 h per day had a higher susceptibility to oesophageal adenocarcinoma compared with those sleeping 7 h [5]. Furthermore, research involving a US cohort identified associations between both short and long sleep durations and the incidence of hepatocellular carcinoma [2]. Additionally, a study in a Spanish cohort linked long sleep duration to an increased risk of having gastric and colorectal cancers [6].

Several mechanisms may account for the link between sleep duration and the risk of developing digestive system cancer. For instance, several nights of short sleep in healthy young men led to changes in the gut microbiota, an imbalance that has been implicated in tumorigenesis [7]. Animal research has furthermore shown that intermittent sleep deprivation in rats compromises gastric mucosal integrity, as evidenced by gastric lesions and increased gastric acidity [8]. Various gastrointestinal functions—such as gastric enzyme and fluid production, nutrient absorption in the small intestine, and gastrointestinal motility—regulate the digestive system’s exposure to carcinogens and follow a distinct circadian rhythm (i.e. a roughly 24-h cycle) [9]. Given the strong connection between sleep and circadian rhythms [10], these functions may be particularly vulnerable to disruption from aberrant sleep duration.

The amount of consumed fibre, fruits, vegetables, and processed foods (such as meat products) may also impact the risk of digestive system cancer. For example, a higher dietary fibre intake has been associated with a lower risk of developing colorectal cancer [11]. Furthermore, increased consumption of fruits and vegetables has shown protective effects against various digestive system cancers, such as oesophageal cancer [12]. In contrast, higher consumption of meat products has been linked to an increased risk of certain cancers, such as gastric cancer [13].

In parallel with these epidemiological findings, it is crucial to consider how dietary factors also impact digestive health. As foods transit through the digestive tract, various factors related to their processing can significantly impact overall digestive health. Key aspects include the level of food processing (e.g. fried vs. raw), as well as additives and the macro- and micronutrient composition of foods. These factors influence not only digestion and nutrient absorption but also the risk of developing or dying from cancers of the digestive system. For instance, as demonstrated in animal models, diets characterised by high-fat content or enriched with food additives—such as colourants, artificial sweeteners, emulsifiers, and nanoparticles—can compromise gut health. These effects may manifest through altered mucus layer production, shifts in the gut microbial landscape from beneficial to pathogenic species, and increased inflammation [14]. Conversely, a high intake of fibre, fruits, and vegetables has been proposed to promote digestive system health. Fibre stimulates the production of short-chain fatty acids, which may have positive effects on gut immunity and increase the abundance of health-promoting species in the gut microbiome [15], as well as contribute to maintain gut barrier integrity [16]. Additionally, fruits and vegetables are rich sources of bioactive compounds like polyphenols. These compounds are thought to enhance gut health by modulating the composition and activity of the colonic microbial population [17].

Whether the protection against cancers of the digestive system conferred by healthy eating habits is less pronounced among individuals with habitual short or long sleep duration remains unclear. Therefore, in this study, we utilized data from over 400,000 participants in the UK Biobank to examine whether the association between healthy dietary patterns—characterized by a high intake of fibre, fruits, and vegetables, alongside low meat consumption—and a reduced risk of mortality from digestive system cancers is diminished or absent in individuals with habitual short or long sleep durations.

## Methods

### Study population and ethics

The present study is a component of the UK Biobank project #80,513. The UK Biobank is a multicentre prospective cohort study that enrolled over 500,000 participants. Recruitment occurred from 2006 to 2010 across 22 assessment centres throughout Great Britain. Baseline data, which included dietary and lifestyle information along with anthropometric measurements, were collected using self-administered questionnaires, computer-assisted interviews, and direct physical measurements. Participants also consented to the linkage of their medical records [18]. Data from 406,584 participants 38 to 73 years of age were used in the present analysis (54.1% women). We employed multiple criteria to define our final cohort, such as withdrawal from the biobank, any cancer at baseline, and missing data for sleep duration, diet components, or covariates, as outlined in Additional file 1: Fig. S1. The UK Biobank study received approval from the North West Multi-centre Research Ethics Committee (ref 11/NW/0382), and all participants provided written informed consent.

### Assessment of sleep duration

As described in previous studies [19–21], habitual sleep duration was assessed based on responses to the question “About how many hours sleep do you get in every 24 h? (please include naps)” from an electronic questionnaire completed during the baseline visit. This question did not specify a particular time frame, and participants were able to indicate their habitual sleep duration in full-hour increments. Participants with a daily sleep duration of 7–8 h were categorised as having normal sleep duration. Short sleep duration was defined as ≤ 6 h per day, while long sleep duration was defined as ≥ 9 h per day. In line with previous research [22], participants with habitual daily sleep durations of less than 3 h or more than 15 h were excluded from the main analysis (Additional file 1: Fig. S1).

### Dietary assessment

Daily fibre intake was evaluated based on participant responses to an electronic questionnaire regarding their consumption of fruits, vegetables, cereals, and bread, with a detailed categorization of bread and cereal types, following the methodology outlined in a previous study using the UK Biobank [23]. Consistent with recommendations [24], a minimum intake of 25 g of fibre per day was considered optimal. Consequently, participants were divided into two groups: those whose intake fell below this threshold (hereafter referred to as “low fibre intake”) and those who met or exceeded it (referred to as “high fibre intake”).

Building upon previous findings indicating a correlation between consuming 7 or more portions of fruits and vegetables daily and reduced cancer mortality [25], individuals consuming fewer than seven pieces of fruits and/or tablespoons of vegetables per day were classified as having a “low intake,” while those consuming seven or more portions were categorized as having a “high intake.”

Additionally, adhering to the recommendation by the World Cancer Research Fund International [26], which advises limiting red and processed meat consumption to approximately three portions per week, participants were segmented into two groups: those with low weekly meat intake (i.e. consuming ≤ 3 servings per week) and those with high weekly meat intake (> 3 servings per week).

Finally, a healthy diet score was constructed based on participants’ adherence to the aforementioned dietary guidelines. Each compliance with a guideline scored one point, with the total healthy diet score ranging from 0 (least healthy) to 3 (most healthy), reflecting the accumulation of healthy eating habits.

### Digestive system cancer death

The date of death was obtained from death certificates through data linkage with national datasets from the) Information Centre (England and Wales) and the NHS Central Register Scotland (Scotland). The International Classification of Diseases, 10th Revision (ICD-10) codes beginning with “C15” to “C26” were employed to identify participants whose primary cause of death was digestive system cancer. The censoring date was 30 September 2021. Participants were followed up from the date of attendance at the recruitment centre (2006–2010) to the date of death or censorship, whichever occurred first. To address reverse causality, death by any cancer events during the first 2 years of follow-up were excluded, resulting in 3 949 deaths due to digestive system cancer during a median follow-up duration of 12.6 years.

### Statistical analyses

All statistical analyses were performed using R v4.4.1 and SPSS version 28.0.1.0 (IBM; SPSS Inc). We employed Cox proportional hazard regression analysis to calculate hazard ratios (HRs) and their 95% confidence interval (CI). Two main Cox regression models were used, selectively incorporating a minimum set of confounding variables to mitigate potential confounding effects and maintain model parsimony. In Model A, a single exposure of interest was considered, which could be either sleep duration or the healthy diet score. Model B incorporated a broader set of variables, including sleep duration, the healthy diet score, age, biological sex (female or male), ethnicity (African or Caribbean, Asian, White, or others), educational level (university degree, no qualification, any other qualification), assessment centre region (England, Wales, or Scotland), socioeconomic status (evaluated using the Townsend index), and frequency of alcohol consumption (non-drinker, less than three times per week, three or more times per week).

We extended Model B by including variables that may lie along the causal pathway from sleep duration and diet to cancer mortality. This included smoking [27–31], where both smoking status (never, former, or current) and pack-years (number of packs per day * number of years smoking) were considered (Model B + Smoking). In a separate analysis, participants’ body mass index (BMI) was added to the regression (Model B + BMI status), given its links with cancer mortality, diet, and sleep [32, 33]. BMI was classified according to World Health Organization guidelines: normal weight (BMI ≥ 18.5 and < 25.0), underweight (BMI < 18.5), overweight (BMI ≥ 25.0 and < 30), obesity class I (BMI ≥ 30.0 and < 35.0), obesity class II (BMI ≥ 35.0 and < 40.0), and obesity class III (BMI ≥ 40). Finally, considering the link between type 2 diabetes (T2D) and increased cancer risk [34], as well as its association with sleep and diet [35–37], a third extended Model B was computed to include T2D incidence before or up to the end of the observational period (Model B + T2D). Details on exposures, outcomes, and covariate data sources within the UK Biobank are provided in Additional file 1: Table S1.

To determine whether the association between healthy eating habits and a lower risk of dying from digestive system cancers might be weaker or absent in individuals with habitual short or long sleep duration, we assessed both multiplicative and additive interactions. For multiplicative interactions, we incorporated terms between sleep duration (three levels) and healthy diet score (four levels) or eating habits (two levels) into Model B. For additive interactions, we calculated the relative excess risk due to interaction (RERI), using the interactionR package version 0.1.7 in R [38]. Sleep duration was categorized as recommended (7–8 h) or outside the recommended range. The healthy diet score was categorized as unhealthy (scores 0 or 1) or healthy (scores 2 and 3), and eating habits were classified as low or high. We then separately evaluated the joint effects of sleep duration and the healthy diet score, as well as each eating habit. In this analysis, binary variables used for the additive interactions were combined, and the reference group was defined as those with sleep duration outside the recommended range and an unhealthy dietary pattern. Given the significance of sex and age as key factors and potential modifiers in epidemiological research, we also assessed multiplicative interactions between sleep duration, healthy diet score, and individual diet components with both sex and age. Additionally, we examined three-way interactions involving sleep duration, healthy diet score or individual diet components, and either sex or age to explore more complex modifying effects.

Four sensitivity analyses were conducted. The first analysis addressed other-cause death as a competing risk for digestive system cancer-specific mortality, employing Fine-Gray subdistribution hazards calculated with the “cmprsk” package version 2.2–12 in R [39]. The second analysis incorporated weekly physical activity levels—categorized as low, moderate, and high according to the International Physical Activity Questionnaire [40]– into Model B. This inclusion was based on previous evidence suggesting that elevated levels of regular physical activity can reduce cancer mortality [41]. To prevent the loss of data for an additional 67,418 participants, missing physical activity data were imputed using the “mice” package version 3.16 in R [42]. We employed proportional odds logistic regression for the imputation and generated five imputed datasets. Cox regression models were then fitted for each dataset, and the results were pooled to obtain combined estimates and standard errors. The potential influence of the imputation process was evaluated by integrating a dummy variable into the model to mark imputed data. In the third sensitivity analysis, participants with a baseline history of the following major non-communicable diseases were excluded prior to analysis: coronary heart disease (I20–I25), stroke (I60–I64), heart failure (I50), chronic obstructive pulmonary disease or asthma exacerbations (J41–J46), and any form of diabetes (E10–E14, O24). To further investigate the potential impact of variations in preferred sleep timing, we conducted a fourth sensitivity analysis incorporating self-reported chronotype (“definitely a morning person,” “more of a morning than an evening person,” “more of an evening than a morning person,” or “definitely an evening person”) into Model B. To avoid the additional exclusion of 40,255 participants, missing chronotype data were imputed using the same method applied to the physical activity data.

Proportional hazard assumptions were verified by assessing Kaplan–Meier survival curves. Interactions and main effects were deemed significant if the *p*-value was less than 0.05.

## Results

### Cohort characteristics

The cohort accrued 5,037,480 years at risk during the follow-up (median follow-up: 12.6 years), with 3949 individuals succumbing to digestive system cancer. Additional File: Table S2 presents the data on deaths caused by digestive system cancer subtypes, categorized by daily sleep duration and healthy diet score.

A total of 68.4% of participants reported a habitual daily sleep duration of 7–8 h, while 24.2% slept 6 h or less, and 7.4% reported sleeping at least 9 h. Fifteen per cent of participants had a diet score of 0 (the lowest score), 42% scored 1, 39.4% scored 2, and 3.6% achieved a score of 3 (the healthiest category). Further details on cohort characteristics, categorized by healthy diet score are presented in Table 1. Cohort characteristics categorized by sleep duration are presented in Additional file 1: Table S3.

**Table 1:** Cohort baseline characteristics categorized by healthy diet score

Characteristic	Healthy diet score				Total
	**0**	**1**	**2**	**3**	
Participants	61,099 (15.0)	170,565 (42.0)	160,233 (39.4)	14,687 (3.6)	406,584
Age, *years*	55.46 ± 8.25	55.88 ± 8.17	56.82 ± 7.91	57.2 ± 7.8	56.23 ± 8.09
Townsend index	− 1.24 ± 3.16	− 1.4 ± 3.03	− 1.51 ± 2.95	− 1.13 ± 3.13	− 1.41 ± 3.02
Daily sleep duration					
* Normal*	41,089 (67.2)	116,488 (68.3)	110,902 (69.2)	9720 (66.2)	278,199 (68.4)
* Short*	15,221 (24.9)	41,128 (24.1)	38,166 (23.8)	3866 (26.3)	98,381 (24.2)
* Long*	4789 (7.8)	12,949 (7.6)	11,165 (7.0)	1101 (7.5)	30,004 (7.4)
Ethnicity					
* White*	58,992 (96.6)	163,523 (95.9)	151,685 (94.7)	13,403 (91.3)	387,603 (95.3)
* Asian*	754 (1.2)	3090 (1.8)	4201 (2.6)	653 (4.4)	8698 (2.1)
* Caribbean or African*	916 (1.5)	2538 (1.5)	2514 (1.6)	283 (1.9)	6251 (1.5)
* Others*	437 (0.7)	1414 (0.8)	1833 (1.1)	348 (2.4)	4032 (1.0)
Educational level					
* No qualification*	10,848 (17.8)	28,284 (16.6)	23,682 (14.8)	2400 (16.3)	65,214 (16.0)
* Any other qualification*	32,101 (52.5)	87,851 (51.5)	78,745 (49.1)	6546 (44.6)	205,243 (50.5)
* University degree*	18,150 (29.7)	54,430 (31.9)	57,806 (36.1)	5741 (39.1)	136,127 (33.5)
Region of the assessment centre					
* England*	52,939 (86.6)	150,839 (88.4)	143,619 (89.6)	13,205 (89.9)	360,602 (88.7)
* Scotland*	2179 (3.6)	6987 (4.1)	6839 (4.3)	580 (3.9)	16,585 (4.1)
* Wales*	5981 (9.8)	12,739 (7.5)	9775 (6.1)	902 (6.1)	29,397 (7.2)
Alcohol consumption					
* Not current*	3612 (5.9)	12,044 (7.1)	12,743 (8.0)	1907 (13.0)	30,306 (7.5)
< *3 times per week*	26,481 (43.3)	82,100 (48.1)	80,472 (50.2)	7629 (51.9)	196,682 (48.4)
≥ *3 times per week*	31,006 (50.7)	76,421 (44.8)	67,018 (41.8)	5151 (35.1)	179,596 (44.2)
Pack-years ^a^	27.63 ± 21.04	23.44 ± 18.38	19.89 ± 16.25	20.3 ± 16.75	22.83 ± 18.34
Smoking status					
* Never*	31,128 (50.9)	94,263 (55.3)	91,910 (57.4)	8280 (56.4)	225,581 (55.5)
* Previous*	20,196 (33.1)	57,599 (33.8)	56,956 (35.5)	5502 (37.5)	140,253 (34.5)
* Current*	9775 (16.0)	18,703 (11.0)	11,367 (7.1)	905 (6.2)	40,750 (10.0)
Type 2 diabetes	5984 (9.8)	14,423 (8.5)	11,638 (7.3)	1302 (8.9)	33,347 (8.2)
BMI status					
* Normal weight*	16,708 (27.3)	53,457 (31.3)	58,151 (36.3)	5616 (38.2)	133,932 (32.9)
* Underweight*	285 (0.5)	816 (0.5)	811 (0.5)	126 (0.9)	2038 (0.5)
* Overweight*	27,105 (44.4)	73,631 (43.2)	66,661 (41.6)	5945 (40.5)	173,342 (42.6)
* Obesity class I*	12,331 (20.2)	30,592 (17.9)	25,007 (15.6)	2145 (14.6)	70,075 (17.2)
* Obesity class II*	3371 (5.5)	8706 (5.1)	6943 (4.3)	602 (4.1)	19,622 (4.8)
* Obesity class III*	1299 (2.1)	3363 (2.0)	2660 (1.7)	253 (1.7)	7575 (1.9)
Physical activity					
* Low*	12,109 (19.8)	29,578 (17.3)	19,882 (12.4)	1324 (9.0)	62,893 (15.5)
* Moderate*	21,216 (34.7)	58,768 (34.5)	54,326 (33.9)	4130 (28.1)	138,440 (34.0)
* High*	17,431 (28.6)	52,481 (30.8)	60,666 (37.9)	7255 (49.4)	137,833 (33.9)
* Missing*	10,343 (16.9)	29,738 (17.4)	25,359 (15.8)	1978 (13.5)	67 418 (16.6)

Data are mean ± SD or *n* (%)

^a^ Pack-years = Number of packs per day multiplied by the number of years smoking

### Sleep duration and digestive system cancer mortality

**Fig. 1:**
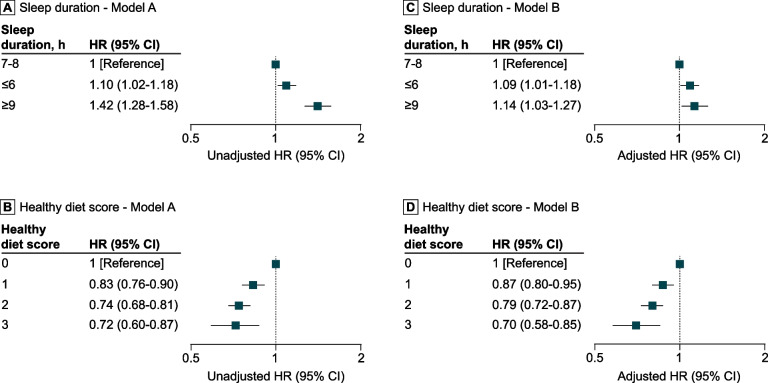
Association of daily sleep duration and healthy diet score with digestive system cancer mortality. Model A (unadjusted) hazard ratios (HR) (95% CI) illustrating the relationship of daily sleep duration (**A**) and the healthy diet score (**B**) with digestive system cancer mortality. Model B (adjusted) HR (95% CI) depicting the relationship of daily sleep duration (**C**) and the healthy diet score (**D**) with digestive system cancer mortality. Model B consisted of variables daily sleep duration, healthy diet score, sex, age, Townsend index, region of assessment centre, ethnicity, educational level, and alcohol consumption frequency

**Fig. 2:**
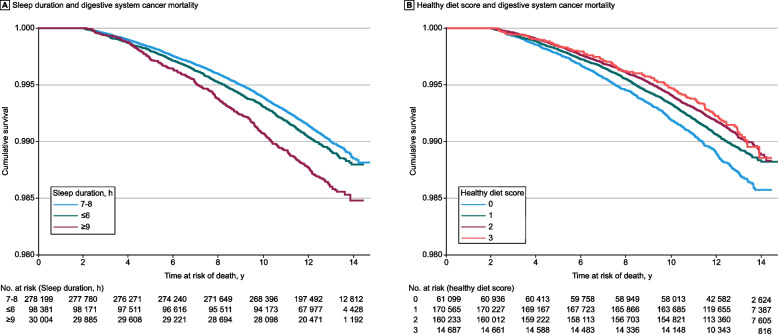
Unadjusted Kaplan–Meier curves showing the relationship of daily sleep duration (**A**) and the healthy diet score (**B**) with digestive system cancer mortality

Compared with individuals reporting 7–8 h of daily sleep (reference), both shorter and longer sleep durations were associated with an increased risk of mortality from digestive system cancers (Model B: HRs, 1.09 (95% CI, 1.01–1.18), *p* = 0.020 for short sleep; 1.14 (95% CI, 1.03–1.27), *p* = 0.015 for long sleep; see Fig. 1, including Model A estimates). Kaplan–Meier curves for the sleep duration groups are depicted in Fig. 2.

**Fig. 3:**
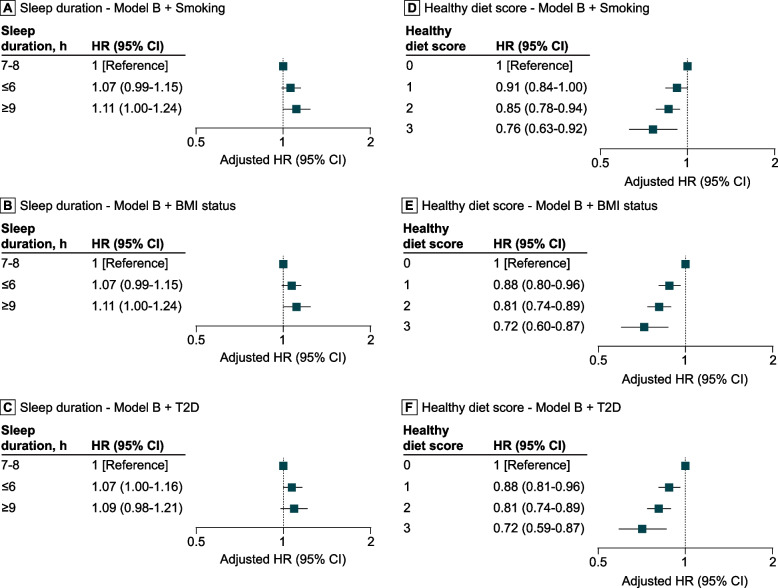
Influence of smoking, weight status and type 2 diabetes on the association of sleep duration and diet with digestive system cancer mortality. Adjusted hazard ratios (HR) (95% CI) depicting the influence of smoking on the relationship of daily sleep duration (**A**) and the healthy diet score (**D**) with digestive system cancer mortality. Adjusted HR (95% CI) illustrating the influence of weight status on the relationship of daily sleep duration (**B**) and the healthy diet score (**E**) with the risk of dying from digestive system cancer. Adjusted HR (95% CI) showing the influence of T2D on the association of daily sleep duration (**C**) and the healthy diet score (**F**) with the risk of succumbing to digestive system cancer. Model B consisted of variables daily sleep duration, healthy diet score, sex, age, Townsend index, region of assessment centre, ethnicity, educational level, and alcohol consumption frequency

The association between sleep duration and digestive system cancer mortality did not reach significance when Model B was additionally adjusted for smoking (HRs, 1.07 (95% CI, 0.99–1.15), *p* = 0.085 for short sleep; 1.11 (95% CI, 1.00–1.24), *p* = 0.060 for long sleep; see Fig. 3), BMI status (HRs, 1.07 (95% CI, 0.99–1.15), *p* = 0.072 for short sleep; 1.11 (95% CI, 1.00–1.24), *p* = 0.058 for long sleep; see Fig. 3), or T2D diagnosis (HRs, 1.07 (95% CI, 1.00–1.16), *p* = 0.059 for short sleep; 1.09 (95% CI, 0.98–1.21), *p* = 0.120 for long sleep; see Fig. 3).

### Healthy diet score and digestive system cancer mortality

Higher healthy diet scores were associated with a reduced risk of digestive system cancer mortality compared with individuals with a healthy diet score of 0 (Model B HRs, 0.87 (95% CI, 0.80–0.95), *p* = 0.002 for score 1; 0.79 (95% CI, 0.72–0.87), *p* < 0.001 for score 2; 0.70 (95% CI, 0.58–0.85), *p* < 0.001 for score 3; see Fig. 1, including Model A estimates). Figure 2 shows Kaplan–Meier curves by healthy diet score.

Consistent results were observed when further adjusting for smoking (HRs, 0.91 (95% CI, 0.84–1.00), *p* = 0.041 for score 1; 0.85 (95% CI, 0.78–0.94), *p* < 0.001 for score 2; 0.76 (95% CI, 0.63–0.92), *p* = 0.006 for score 3; see Fig. 3), BMI status (HRs, 0.88 (95% CI, 0.80–0.96), *p* = 0.004 for score 1; 0.81 (95% CI, 0.74–0.89), *p* < 0.001 for score 2; 0.72 (95% CI, 0.60–0.87), *p* < 0.001 for score 3; see Fig. 3), or T2D diagnosis (HRs, 0.88 (95% CI, 0.81–0.96), *p* = 0.004 for score 1; 0.81 (95% CI, 0.74–0.89), *p* < 0.001 for score 2; 0.72 (95% CI, 0.59–0.87), *p* < 0.001 for score 3; see Fig. 3). Additional file 1: Table S4 presents the HRs for each dietary habit. Notably, a high daily intake of fruits and vegetables, along with low weekly meat consumption, was significantly associated with a lower risk of dying from digestive system cancers (*P* ≤ 0.032). In contrast, no significant protection was observed for a high intake of fibre (*P* ≥ 0.098).

### Interaction analyses and joint effects between sleep duration and diet

None of the multiplicative interaction terms between sleep duration and healthy diet score or individual eating habits reached statistical significance. Specifically, *p*-values were 0.599 for sleep duration * healthy diet score; 0.334 for sleep duration * fruits and vegetables intake; 0.063 for sleep duration * meat intake; and 0.820 for sleep duration * fibre intake. Two- and three-way interaction terms between sleep duration, healthy diet score, or diet components and sex or age were not statistically significant (*P* ≥ 0.091; detailed values are provided in Additional file 1: Table S5). Additionally, interaction terms between sleep duration and the healthy diet score, or individual eating habits, remained non-significant in subgroup analyses stratified by age (< 65 years: *P* ≥ 0.188; ≥ 65 years: *P* ≥ 0.232) and sex (men: *P* ≥ 0.113; women: *P* ≥ 0.233). Different sexes and age groups estimates (< 65 years and ≥ 65 years) are shown in Additional file 1: Fig. S2.

In the next step, additive interactions were tested in the full cohort. The RERIs were 0.06 (95% CI, − 0.09 to 0.21) for sleep duration + healthy diet score; 0.06 (95% CI, −0.09 to 0.21) for sleep duration + fruit and vegetable intake; − 0.02 (95% CI, − 0.18 to 0.14) for sleep duration + meat intake; and − 0.03 (95% CI, − 0.39 to 0.32) for sleep duration + fibre intake. Table 2 displays the joint effects of sleep duration and diet on digestive system cancer mortality.

**Table 2:** Joint effects of sleep duration and diet on digestive cancer mortality

			**Joint analysis**		**Stratified analysis**	
**Sleep duration**	**Diet**	***N***	**HR (95% CI)**	***P*-value**	**HR (95% CI)**	***P*-value**
Recommended	Healthy diet	120,622	0.78 (0.71–0.86)	< 0.001	0.86 (0.77–0.97)	< 0.001
	Unhealthy diet	157,577	0.89 (0.82–0.97)	0.007	Ref	
Outside recommended range	Healthy diet	54,298	0.84 (0.76–0.94)	0.003	0.87 (0.80–0.94)	< 0.001
	Unhealthy diet	74,087	Ref		Ref	
Recommended	Fruits and vegetables: high intake	161,158	0.79 (0.72–0.87)	< 0.001	0.88 (0.79–0.98)	0.002
	Fruits and vegetables: low intake	117,041	0.88 (0.80–0.98)	0.015	Ref	
Outside recommended range	Fruits and vegetables: high intake	73,374	0.86 (0.77–0.95)	0.005	0.88 (0.82–0.96)	0.002
	Fruits and vegetables: low intake	55,011	Ref		Ref	
Recommended	Meat: low intake	193,513	0.81 (0.73–0.90)	< 0.001	0.92 (0.83–1.03)	0.163
	Meat: high intake	84,686	0.92 (0.83–1.03)	0.134	Ref	
Outside recommended range	Meat: low intake	87,576	0.90 (0.81–1.01)	0.072	0.87 (0.80–0.95)	0.001
	Meat: high intake	40,809	Ref		Ref	
Recommended	Fibre: high intake	12,781	0.74 (0.60–0.90)	0.002	0.85 (0.67–1.09)	0.207
	Fibre: low intake	265,418	0.91 (0.85–0.97)	0.004	Ref	
Outside recommended range	Fibre: high intake	6690	0.86 (0.67–1.10)	0.220	0.82 (0.67–0.99)	0.041
	Fibre: low intake	121,695	Ref		Ref	

### Sensitivity analyses

The Fine-Gray proportional hazards regression analysis, which accounted for other causes of death as a competing risk, largely supported our main findings about the relationship of sleep duration and diet with the risk of dying from digestive system cancer (HRs, 1.09 (95% CI, 1.01–1.17), *p* = 0.029 for short sleep; 1.12 (95% CI, 1.01–1.25), *p* = 0.039 for long sleep; 0.88 (95% CI, 0.80–0.96), *p* = 0.004 for healthy diet score 1; 0.80 (95% CI, 0.73–0.88), *p* < 0.001 for score 2; and 0.71 (95% CI, 0.59–0.86), *p* < 0.001 for score 3).

When physical activity levels were incorporated into Model B, the primary results were corroborated. Short and long sleep durations were associated with an increased risk of succumbing to digestive system cancer (HR, 1.09 (95% CI, 1.01–1.17), *p* = 0.024 for short sleep; HR, 1.14 (95% CI, 1.02–1.26), *p* = 0.021 for long sleep), while adherence to a healthy dietary pattern continued to show a protective effect (HRs, 0.88 (95% CI, 0.80–0.96), *p* = 0.003 for score 1; 0.81 (95% CI, 0.74–0.89), *p* < 0.001 for score 2; and 0.72 (95% CI, 0.60–0.88), *p* < 0.001 for score 3).

In the third sensitivity analysis, which excluded participants with a baseline history of major non-communicable diseases, habitual long sleep duration (but not short sleep duration) remained associated with an increased risk of mortality from digestive system cancer (HR, 1.04 (95% CI, 0.95–1.13), *p* = 0.425 for short sleep; HR, 1.17 (95% CI, 1.03–1.34), *p* = 0.019 for long sleep). Similar to the findings in the main analysis, diet scores greater than 0 were associated with a reduced risk of mortality from digestive system cancers (HRs, 0.85 (95% CI, 0.76–0.94), *p* = 0.003 for score 1; 0.81 (95% CI, 0.72–0.90), *p* < 0.001 for score 2; and 0.69 (95% CI, 0.55–0.87), *p* = 0.002 for score 3).

Finally, the primary results were confirmed after incorporating self-reported chronotype into Model B. Short and long sleep durations remained associated with an increased risk of mortality from digestive system cancer (HR, 1.08 (95% CI, 1.01–1.17), *p* = 0.038 for short sleep; HR, 1.12 (95% CI, 1.01–1.25), *p* = 0.049 for long sleep). Additionally, adherence to a healthy dietary pattern continued to exhibit a protective effect (HRs, 0.86 (95% CI, 0.78–0.94), *p* = 0.001 for score 1; 0.78 (95% CI, 0.71–0.86), *p* < 0.001 for score 2; and 0.69 (95% CI, 0.57–0.84), *p* < 0.001 for score 3).

## Discussion

Using data from a large cohort, the present study found that a healthy dietary pattern—characterised by a high intake of fibre (> 25 g per day), fruits, and vegetables (≥ 7 portions per day), and low meat consumption (≤ 3 servings per week)—was associated with a lower risk of dying from digestive system cancer over a follow-up period of approximately 13 years. Conversely, daily sleep duration below or above 7 to 8 h was associated with an increased risk. Public health strategies that promote sleeping 7 to 8 h per day and adhering to diets rich in fibre, fruits, and vegetables while low in meat may therefore be effective in reducing the risk of dying from digestive cancers.

The observation that short and long sleep duration remained associated with digestive system cancer mortality even after adjusting for the healthy diet score (in Model B, see results) suggests that food choices are unlikely to explain the observed association of sleep duration with cancer mortality. Previous studies have linked both short and long sleep duration with increased risks of obesity (e.g. due to higher energy intake associated with short sleep duration or physical inactivity resulting from habitual long sleep duration), T2D, a higher likelihood of smoking, and sedentary lifestyle [30–32, 36, 37, 43–45]. In the present study, participants with both short and long sleep durations (outside the 7–8 h range) were more likely to have a BMI ≥ 30 kg/m^2^, T2D, report past or current smoking, and were more frequently physically less active. Given that smoking, high BMI (i.e. obesity), T2D, and a sedentary lifestyle have all been linked to an increased risk of cancer [46–49], it could be hypothesized that these factors may account for the observed associations between aberrant sleep durations and mortality from digestive system cancers. Supporting this assumption, the inclusion of BMI, T2D, and smoking—but not physical activity—resulted in the loss of significance for the association between aberrant sleep durations and mortality from digestive system cancer. This suggests that these factors may play a more relevant role than physical activity along the causal pathway between sleep duration and digestive system cancer mortality. Additionally, the observation that habitual short daily sleep duration was no longer associated with an increased risk of mortality from digestive system cancer after excluding participants with a baseline history of major non-communicable diseases further suggests that short sleep duration may be a consequence of these pre-existing health conditions, rather than an independent risk factor for cancer mortality.

Although healthy eating, as defined in this study, may offer some protection against higher body weight and T2D [35, 50–52], and may be associated with non-smoking due to increased health awareness, adjusting for these factors did not change the association between healthy eating and the cancer outcome. This suggests that the benefits of a healthy diet on cancer risk are likely due to direct effects on digestive tract health, which is not surprising given that food directly interacts with and is processed by the digestive system.

Given that habitual short and long sleep duration may be unavoidable due to life circumstances (e.g. parenting, commuting, and health conditions), making healthy dietary choices can be an alternative strategy for promoting health. Our findings suggest that, at least in the context of digestive system cancer mortality, maintaining a healthy diet characterised by a high intake of fibre, fruits, and vegetables, along with low meat consumption, can reduce this risk across all investigated sleep duration groups. Therefore, prioritising a healthy diet remains a promising component of the prevention of cancers afflicting the digestive system, regardless of sleep patterns.

Due to the nature of our study, we cannot establish causal inferences. Nonetheless, as mentioned earlier, both sleep duration and the dietary habits investigated have been shown to affect the health of the digestive tract through various mechanisms, including impacts on the gut microbiome, gut barrier function, inflammation, and food transit time [1–3, 10–12]. Additionally, food processing, such as such as curing and smoking of meat, can result in the formation of carcinogenic chemicals, including N-nitroso-compounds and polycyclic aromatic hydrocarbons [53].

## Limitations

While our findings remained robust across sensitivity analyses, it is important to acknowledge several limitations. We focused on subjective daily sleep duration, which may be susceptible to recall bias. Although subjective sleep duration may not perfectly align with objective measurements, it offers practical applicability in a clinical context by relying on a simple question. Compared with subjective sleep assessments, objective measures of sleep duration, such as home or laboratory sleep monitoring, may also have limitations due to potential bias from participants’ heightened vigilance resulting from increased focus on sleep. Another advantage of using subjective instead of objective sleep duration, as evaluated through accelerometry in the UK Biobank, is that the latter was only available for a subset of UK participants (approximately one-fifth), potentially limiting the exploration of the interplay between habitual sleep duration, diet, and digestive system cancer mortality.

Another limitation of our study is the lack of updated data on follow-up unavailability from the UK Biobank beyond May 2017. Our study population was also mainly composed of individuals with white ethnicity, which may hinder the applicability of our results to other ethnical groups. Although no interaction was found between dietary behaviours and sleep duration on digestive cancer mortality risk, adherence to other healthy lifestyle factors known to reduce this risk (e.g. regular physical activity; [49]) may lower the risk in individuals with poor diet or sleep habits outside the recommended range. Likewise, it cannot be ruled out that dietary patterns not examined in our study, such as time-restricted eating or Mediterranean diet (e.g. [54, 55]), may not only protect against digestive system cancer but also mitigate the risk associated with habitual sleep durations that fall outside the recommended range for adults.

Another limitation is the potential for changes in dietary patterns over time. However, a separate UK Biobank study addressed this by evaluating the reproducibility of the baseline dietary assessment in a sub-cohort of 20,000 participants who completed a 24-h dietary recall four years later. For key food groups (fruit, vegetables, fish, meat) and the partial fibre score, there was moderate to substantial agreement between baseline and follow-up assessments [23].

When interpreting the findings of the present study, it is important to note that the outcome encompassed all cancers within the digestive system that resulted in mortality. Survival times can vary significantly between different cancer sites within the digestive system [56]. As such, it cannot be ruled out that the extent to which habitual sleep duration and dietary habits independently or interactively influence the risk of digestive system cancer mortality may be site-specific. Additionally, variations in cancer treatment efficacy and patients’ therapeutic responsiveness, which were not accounted for in our study, may further complicate the interpretation of these findings.

## Conclusions

Short and long sleep duration may elevate the risk of mortality from digestive system cancer, potentially through pathways involving smoking, high BMI, and T2D. However, short sleep duration could also be a consequence of pre-existing non-communicable diseases, rather than an independent risk factor. The protective effects of healthy dietary patterns seem to remain preserved regardless of sleep duration. These findings suggest that public health strategies should emphasise the importance of maintaining a healthy diet rich in fibre, fruits, and vegetables, along with low meat consumption, as a means of mitigating cancer risk, even among those with non-ideal sleep patterns. However, it is important to consider the limitations of this study, and further research is needed to validate these findings and explore the potential interactions between lifestyle factors in diverse populations, including cancer-specific cohorts, where the effects of dietary habits and sleep duration may vary.

## Supplementary Information

Additional file 1. Table S1. Source of exposure, outcomes, and covariates within the UK Biobank. Table S2. Deaths caused by digestive system cancer subtype categorized by daily sleep duration and healthy diet score. Table S3. Cohort baseline characteristics categorized by daily sleep duration. Table S4. Effects of eating habits on digestive organs cancer mortality. Table S5. Multiplicative interactions with sex and age. Fig. S1. STROBE diagram. Fig. S2. Association of daily sleep duration and healthy diet score with digestive system cancer mortality, stratified by sex and age

## Data Availability

No datasets were generated or analysed during the current study.

## Abbreviations

HR: Hazard ratio
CI: Confidence interval
T2D: Type 2 diabetes
BMI: Body mass index
RERI: Relative excess risk due to interaction
NHS: National Health Service
ICD-10: The International Classification of Diseases, 10th Revision

## References

[1] Arnold M, Abnet CC, Neale RE, Vignat J, Giovannucci EL, McGlynn KA, et al. Global Burden of 5 Major Types of Gastrointestinal Cancer. Gastroenterology. 2020;159:335-349.e15. 32247694 10.1053/j.gastro.2020.02.068 PMC8630546

[2] Long L, Zhao L, Petrick JL, Liao LM, Huang T, Hakim A, et al. Daytime napping, nighttime sleeping duration, and risk of hepatocellular carcinoma and liver disease-related mortality. JHEP Rep Innov Hepatol. 2023;5: 100819. 10.1016/j.jhepr.2023.100819 PMC10482745 37691690

[3] Collatuzzo G, Pelucchi C, Negri E, Kogevinas M, Huerta JM, Vioque J, et al. Sleep Duration and Stress Level in the Risk of Gastric Cancer: A Pooled Analysis of Case-Control Studies in the Stomach Cancer Pooling (StoP) Project. Cancers. 2023;15:4319. 37686594 10.3390/cancers15174319 PMC10486543

[4] Wang G, Wang J-J, Lin C-H, Zhou Q, Wang W-L, Qin T, et al. Association of sleep duration, sleep apnea, and shift work with risk of colorectal neoplasms: a systematic review and meta-analysis. J Gastrointest Oncol. 2022;13:1805–17. 36092341 10.21037/jgo-22-682 PMC9459215

[5] Wang X, Tian R, Zong X, Jeon MS, Luo J, Colditz GA, et al. Sleep Behaviors, Genetic Predispositions, and Risk of Esophageal Cancer. Cancer Epidemiol Biomark Prev Publ Am Assoc Cancer Res Cosponsored Am Soc Prev Oncol. 2023;32:1079–86. 10.1158/1055-9965.EPI-23-0101 PMC10525008 37195052

[6] Papantoniou K, Castaño-Vinyals G, Espinosa A, Turner MC, Martín-Sánchez V, Casabonne D, et al. Sleep duration and napping in relation to colorectal and gastric cancer in the MCC-Spain study. Sci Rep. 2021;11:11822. 34083698 10.1038/s41598-021-91275-3 PMC8175745

[7] Benedict C, Vogel H, Jonas W, Woting A, Blaut M, Schürmann A, et al. Gut microbiota and glucometabolic alterations in response to recurrent partial sleep deprivation in normal-weight young individuals. Mol Metab. 2016;5:1175–86. 27900260 10.1016/j.molmet.2016.10.003 PMC5123208

[8] Guo JS, Chau JFL, Cho CH, Koo MWL. Partial sleep deprivation compromises gastric mucosal integrity in rats. Life Sci. 2005;77:220–9. 15862606 10.1016/j.lfs.2004.12.027

[9] Duboc H, Coffin B, Siproudhis L. Disruption of Circadian Rhythms and Gut Motility: An Overview of Underlying Mechanisms and Associated Pathologies. J Clin Gastroenterol. 2020;54:405–14. 32134798 10.1097/MCG.0000000000001333 PMC7147411

[10] Cedernaes J, Osler ME, Voisin S, Broman J-E, Vogel H, Dickson SL, et al. Acute Sleep Loss Induces Tissue-Specific Epigenetic and Transcriptional Alterations to Circadian Clock Genes in Men. J Clin Endocrinol Metab. 2015;100:E1255-1261. 26168277 10.1210/JC.2015-2284

[11] Ben Q, Sun Y, Chai R, Qian A, Xu B, Yuan Y. Dietary fiber intake reduces risk for colorectal adenoma: a meta-analysis. Gastroenterology. 2014;146:689-699.e6. 24216326 10.1053/j.gastro.2013.11.003

[12] Steevens J, Schouten LJ, Goldbohm RA, van den Brandt PA. Vegetables and fruits consumption and risk of esophageal and gastric cancer subtypes in the Netherlands Cohort Study. Int J Cancer. 2011;129:2681–93. 21960262 10.1002/ijc.25928

[13] Ferro A, Rosato V, Rota M, Costa AR, Morais S, Pelucchi C, et al. Meat intake and risk of gastric cancer in the Stomach cancer Pooling (StoP) project. Int J Cancer. 2020;147:45–55. 31584199 10.1002/ijc.32707 PMC8550819

[14] Srour B, Kordahi MC, Bonazzi E, Deschasaux-Tanguy M, Touvier M, Chassaing B. Ultra-processed foods and human health: from epidemiological evidence to mechanistic insights. Lancet Gastroenterol Hepatol. 2022;7:1128–40. 35952706 10.1016/S2468-1253(22)00169-8

[15] Slavin J. Fiber and prebiotics: mechanisms and health benefits. Nutrients. 2013;5:1417–35. 23609775 10.3390/nu5041417 PMC3705355

[16] Kelly CJ, Zheng L, Campbell EL, Saeedi B, Scholz CC, Bayless AJ, et al. Crosstalk between Microbiota-Derived Short-Chain Fatty Acids and Intestinal Epithelial HIF Augments Tissue Barrier Function. Cell Host Microbe. 2015;17:662–71. 25865369 10.1016/j.chom.2015.03.005 PMC4433427

[17] Cardona F, Andrés-Lacueva C, Tulipani S, Tinahones FJ, Queipo-Ortuño MI. Benefits of polyphenols on gut microbiota and implications in human health. J Nutr Biochem. 2013;24:1415–22. 23849454 10.1016/j.jnutbio.2013.05.001

[18] Sudlow C, Gallacher J, Allen N, Beral V, Burton P, Danesh J, et al. UK biobank: an open access resource for identifying the causes of a wide range of complex diseases of middle and old age. PLoS Med. 2015;12: e1001779. 25826379 10.1371/journal.pmed.1001779 PMC4380465

[19] Xie J, Zhu M, Ji M, Fan J, Huang Y, Wei X, et al. Relationships between sleep traits and lung cancer risk: a prospective cohort study in UK Biobank. Sleep. 2021;44:zsab089. 10.1093/sleep/zsab089 33823024

[20] Freeman JR, Saint-Maurice PF, Zhang T, Matthews CE, Stolzenberg-Solomon RZ. Sleep and Risk of Pancreatic Cancer in the UK Biobank. Cancer Epidemiol Biomark Prev Publ Am Assoc Cancer Res Cosponsored Am Soc Prev Oncol. 2024;33:624–7. 10.1158/1055-9965.EPI-23-0983 PMC10990775 38387085

[21] Titova OE, Michaëlsson K, Vithayathil M, Mason AM, Kar S, Burgess S, et al. Sleep duration and risk of overall and 22 site-specific cancers: A Mendelian randomization study. Int J Cancer. 2021;148:914–20. 32895918 10.1002/ijc.33286 PMC7821333

[22] Xue P, Merikanto I, Chung F, Morin CM, Espie C, Bjorvatn B, et al. Persistent short nighttime sleep duration is associated with a greater post-COVID risk in fully mRNA-vaccinated individuals. Transl Psychiatry. 2023;13:32. 36726008 10.1038/s41398-023-02334-4 PMC9890416

[23] Bradbury KE, Young HJ, Guo W, Key TJ. Dietary assessment in UK Biobank: an evaluation of the performance of the touchscreen dietary questionnaire. J Nutr Sci. 2018;7: e6. 29430297 10.1017/jns.2017.66 PMC5799609

[24] Increasing Fiber Intake. ucsfhealth.org. https://www.ucsfhealth.org/education/increasing-fiber-intake. Accessed 15 Aug 2024.

[25] Oyebode O, Gordon-Dseagu V, Walker A, Mindell JS. Fruit and vegetable consumption and all-cause, cancer and CVD mortality: analysis of Health Survey for England data. J Epidemiol Community Health. 2014;68:856–62. 24687909 10.1136/jech-2013-203500 PMC4145465

[26] Limit red and processed meat. WCRF International. https://www.wcrf.org/diet-activity-and-cancer/cancer-prevention-recommendations/limit-red-and-processed-meat/. Accessed 15 Aug 2024.

[27] Islami F, Marlow EC, Thomson B, McCullough ML, Rumgay H, Gapstur SM, et al. Proportion and number of cancer cases and deaths attributable to potentially modifiable risk factors in the United States, 2019. CA Cancer J Clin. 2024. 10.3322/caac.21858. 38990124 10.3322/caac.21858

[28] Patterson F, Grandner MA, Lozano A, Satti A, Ma G. Transitioning from adequate to inadequate sleep duration associated with higher smoking rate and greater nicotine dependence in a population sample. Addict Behav. 2018;77:47–50. 28950118 10.1016/j.addbeh.2017.09.011 PMC5701829

[29] Boakye D, Wyse CA, Morales-Celis CA, Biello SM, Bailey MES, Dare S, et al. Tobacco exposure and sleep disturbance in 498 208 UK Biobank participants. J Public Health Oxf Engl. 2018;40:517–26. 10.1093/pubmed/fdx102 PMC6166587 29040744

[30] Larson NI, Story M, Perry CL, Neumark-Sztainer D, Hannan PJ. Are diet and physical activity patterns related to cigarette smoking in adolescents? Findings from Project EAT. Prev Chronic Dis. 2007;4:A51. 17572955 PMC1955390

[31] Alkerwi A, Baydarlioglu B, Sauvageot N, Stranges S, Lemmens P, Shivappa N, et al. Smoking status is inversely associated with overall diet quality: Findings from the ORISCAV-LUX study. Clin Nutr Edinb Scotl. 2017;36:1275–82. 10.1016/j.clnu.2016.08.013 27595637

[32] Cappuccio FP, Taggart FM, Kandala N-B, Currie A, Peile E, Stranges S, et al. Meta-analysis of short sleep duration and obesity in children and adults. Sleep. 2008;31:619–26. 18517032 10.1093/sleep/31.5.619 PMC2398753

[33] Calle EE, Rodriguez C, Walker-Thurmond K, Thun MJ. Overweight, obesity, and mortality from cancer in a prospectively studied cohort of U.S. adults. N Engl J Med. 2003;348:1625–38. 10.1056/NEJMoa021423 12711737

[34] Pearson-Stuttard J, Papadimitriou N, Markozannes G, Cividini S, Kakourou A, Gill D, et al. Type 2 Diabetes and Cancer: An Umbrella Review of Observational and Mendelian Randomization Studies. Cancer Epidemiol Biomark Prev Publ Am Assoc Cancer Res Cosponsored Am Soc Prev Oncol. 2021;30:1218–28. 10.1158/1055-9965.EPI-20-1245 PMC9398112 33737302

[35] Neuenschwander M, Ballon A, Weber KS, Norat T, Aune D, Schwingshackl L, et al. Role of diet in type 2 diabetes incidence: umbrella review of meta-analyses of prospective observational studies. BMJ. 2019;366: l2368. 31270064 10.1136/bmj.l2368 PMC6607211

[36] Nôga DA, Meth E de MES, Pacheco AP, Tan X, Cedernaes J, van Egmond LT, et al. Habitual Short Sleep Duration, Diet, and Development of Type 2 Diabetes in Adults. JAMA Netw Open. 2024;7:e241147. 10.1001/jamanetworkopen.2024.1147 PMC10915681 38441893

[37] Shan Z, Ma H, Xie M, Yan P, Guo Y, Bao W, et al. Sleep duration and risk of type 2 diabetes: a meta-analysis of prospective studies. Diabetes Care. 2015;38:529–37. 25715415 10.2337/dc14-2073

[38] Alli BY. InteractionR: An R package for full reporting of effect modification and interaction. Softw Impacts. 2021;10: 100147.

[39] Gray RJ. A Class of K-Sample Tests for Comparing the Cumulative Incidence of a Competing Risk. Ann Stat. 1988;16:1141–54.

[40] Craig CL, Marshall AL, Sjöström M, Bauman AE, Booth ML, Ainsworth BE, et al. International physical activity questionnaire: 12-country reliability and validity. Med Sci Sports Exerc. 2003;35:1381–95. 12900694 10.1249/01.MSS.0000078924.61453.FB

[41] Huang B-H, Duncan MJ, Cistulli PA, Nassar N, Hamer M, Stamatakis E. Sleep and physical activity in relation to all-cause, cardiovascular disease and cancer mortality risk. Br J Sports Med. 2022;56:718–24. 34187783 10.1136/bjsports-2021-104046

[42] van Buuren S, Groothuis-Oudshoorn K. mice: Multivariate Imputation by Chained Equations in R. J Stat Softw. 2011;45:1–67.

[43] Brondel L, Romer MA, Nougues PM, Touyarou P, Davenne D. Acute partial sleep deprivation increases food intake in healthy men. Am J Clin Nutr. 2010;91:1550–9. 20357041 10.3945/ajcn.2009.28523

[44] Tan X, Chapman CD, Cedernaes J, Benedict C. Association between long sleep duration and increased risk of obesity and type 2 diabetes: A review of possible mechanisms. Sleep Med Rev. 2018;40:127–34. 29233612 10.1016/j.smrv.2017.11.001

[45] Schmid SM, Hallschmid M, Jauch-Chara K, Wilms B, Benedict C, Lehnert H, et al. Short-term sleep loss decreases physical activity under free-living conditions but does not increase food intake under time-deprived laboratory conditions in healthy men. Am J Clin Nutr. 2009;90:1476–82. 19846546 10.3945/ajcn.2009.27984

[46] Giovannucci E, Harlan DM, Archer MC, Bergenstal RM, Gapstur SM, Habel LA, et al. Diabetes and cancer: a consensus report. Diabetes Care. 2010;33:1674–85. 20587728 10.2337/dc10-0666 PMC2890380

[47] Harris BHL, Macaulay VM, Harris DA, Klenerman P, Karpe F, Lord SR, et al. Obesity: a perfect storm for carcinogenesis. Cancer Metastasis Rev. 2022;41:491–515. 36038791 10.1007/s10555-022-10046-2 PMC9470699

[48] Caliri AW, Tommasi S, Besaratinia A. Relationships among smoking, oxidative stress, inflammation, macromolecular damage, and cancer. Mutat Res Rev Mutat Res. 2021;787: 108365. 34083039 10.1016/j.mrrev.2021.108365 PMC8287787

[49] Xie F, You Y, Huang J, Guan C, Chen Z, Fang M, et al. Association between physical activity and digestive-system cancer: An updated systematic review and meta-analysis. J Sport Health Sci. 2021;10:4–13. 33010525 10.1016/j.jshs.2020.09.009 PMC7856558

[50] Waddell IS, Orfila C. Dietary fiber in the prevention of obesity and obesity-related chronic diseases: From epidemiological evidence to potential molecular mechanisms. Crit Rev Food Sci Nutr. 2023;63:8752–67. 35471164 10.1080/10408398.2022.2061909

[51] Becerra-Tomás N, Díaz-López A, Rosique-Esteban N, Ros E, Buil-Cosiales P, Corella D, et al. Legume consumption is inversely associated with type 2 diabetes incidence in adults: A prospective assessment from the PREDIMED study. Clin Nutr. 2018;37:906–13. 28392166 10.1016/j.clnu.2017.03.015

[52] Zhao L, Zhang F, Ding X, Wu G, Lam YY, Wang X, et al. Gut bacteria selectively promoted by dietary fibers alleviate type 2 diabetes. Science. 2018;359:1151–6. 29590046 10.1126/science.aao5774

[53] Bouvard V, Loomis D, Guyton KZ, Grosse Y, Ghissassi FE, Benbrahim-Tallaa L, et al. Carcinogenicity of consumption of red and processed meat. Lancet Oncol. 2015;16:1599–600. 26514947 10.1016/S1470-2045(15)00444-1

[54] Giacosa A, Barale R, Bavaresco L, Gatenby P, Gerbi V, Janssens J, et al. Cancer prevention in Europe: the Mediterranean diet as a protective choice. Eur J Cancer Prev Off J Eur Cancer Prev Organ ECP. 2013;22:90–5. 10.1097/CEJ.0b013e328354d2d7 22644232

[55] Das M, Webster NJG. Obesity, cancer risk, and time-restricted eating. Cancer Metastasis Rev. 2022;41:697–717. 35984550 10.1007/s10555-022-10061-3 PMC9470651

[56] Klint A, Engholm G, Storm HH, Tryggvadóttir L, Gislum M, Hakulinen T, et al. Trends in survival of patients diagnosed with cancer of the digestive organs in the Nordic countries 1964–2003 followed up to the end of 2006. Acta Oncol Stockh Swed. 2010;49:578–607. 10.3109/02841861003739330 20491524

