# Supplementary material for: Habitual sleep duration, healthy eating, and digestive system cancer mortality
Source: BMC Med. 2025 Jan 27;23:44. doi: 10.1186/s12916-025-03882-w (PMC11770963; doi:10.1186/s12916-025-03882-w)
Supplement: Supplementary file 1 — Additional file 1. Table S1. Source of exposure, outcomes, and covariates within the UK Biobank. Table S2. Deaths caused by digestive system cancer subtype categorized by daily sleep duration and healthy diet score. Table S3. Cohort baseline characteristics categorized by daily sleep duration. Table S4. Effects of eating habits on digestive organs cancer mortality. Table S5. Multiplicative interactions with sex and age. Fig. S1. STROBE diagram. Fig. S2. Association of daily sleep duration and healthy diet score with digestive system cancer mortality, stratified by sex and age [file 12916_2025_3882_MOESM1_ESM.pdf]

Supplemental material: Habitual Sleep Duration, Healthy Eating, and Digestive System Cancer Mortality

Diana A. Nôga, Elisa M. S. Meth, André P. Pacheco, Jonathan Cedernaes, Pei Xue, Christian Benedict

Table of contents

Table S1. Source of exposure, outcomes and covariates within the UK Biobank 2
Table S2. Deaths caused by digestive system cancer subtype categorized by daily sleep duration and healthy diet score 3
Table S3. Cohort baseline characteristics categorized by daily sleep duration 4
Table S4. Effects of eating habits on digestive organs cancer mortality 5
Table S5. Multiplicative interactions with sex and age 5
Figure S1. STROBE diagram 6
Figure S2. Association of daily sleep duration and healthy diet score with digestive system cancer mortality stratified by sex and age 7

Table S1. Source of exposure, outcomes and covariates within the UK Biobank

Variable	UK Biobank field ID
Primary cause of death	40001
Date of death	40000
Date of baseline assessment	53
Sleep duration	1160
Meat intake	1349, 1389, 1379, and 1369
Fruits and Vegetables intake	1309, 1319, 1289, and 1299
Fibre intake	1309, 1319, 1289, 1299, 1448, 1438, 1468 and 1458
Age	21022
Sex	31
Townsend index	22189
Race and Ethnicity	21000
Educational level	6138
Region of assessment center	54
Alcohol consumption frequency	1558
Pack years	20161
Smoking status	20116
Weight status (BMI)	21001
T2D diagnosis	130708
Physical activity level	22032
Cancer at baseline	41270, 40006, 41280, 40005 (ICD-10 “C00-C97”)
Major non-communicable diseases	130706, 130708, 130710, 130712, 130714, 132202 (any diabetes, E10-14, O24)
	131486, 131488, 131490, 131492, 131494, 131496 (COPD or asthma exacerbations, J41-46)
	131296, 131298, 131300, 131302, 131304, 131306 (Coronary heart disease, I20-25)
	131360, 131362, 131364, 131366, 131368 (Stroke, I60-64)
	131354 (Heart failure, I50)
Chronotype	1180

COPD = chronic obstructive pulmonary disease.

Table S2. Deaths caused by digestive system cancer subtype categorized by daily sleep duration and healthy diet score

Cancer subtype (ICD-10)	Daily sleep duration			Healthy diet score				Total
	Normal	Short	Long	0	1	2	3	
Esophagus (C15)	376 (0.14)	157 (0.16)	68 (0.22)	144 (0.24)	272 (0.16)	166 (0.10)	19 (0.13)	601 (0.14)
Stomach (C16)	200 (0.07)	89 (0.09)	22 (0.07)	58 (0.09)	143 (0.08)	99 (0.06)	11 (0.07)	311 (0.08)
Small intestine (C17)	33 (0.01)	9 (0.01)	4 (0.01)	6 (0.01)	20 (0.01)	20 (0.01)	0 (0)	46 (0.01)
Colorectum (C18-20)	811 (0.29)	289 (0.29)	114 (0.38)	220 (0.36)	499 (0.29)	452 (0.28)	43 (0.29)	1214 (0.30)
Anus (C21)	19 (<0.01)	10 (0.01)	3 (0.01)	5 (0.01)	9 (<0.01)	16 (0.01)	2 (0.01)	32 (<0.01)
Liver (C22)	281 (0.10)	124 (0.12)	54 (0.18)	90 (0.15)	200 (0.12)	155 (0.10)	14 (0.09)	459 (0.11)
Gallbladder (C23)	34 (0.01)	15 (0.02)	10 (0.03)	5 (0.01)	22 (0.01)	32 (0.02)	0 (0)	59 (0.01)
Biliary tract (C24)	24 (<0.01)	12 (0.01)	3 (0.01)	7 (0.01)	13 (<0.01)	17 (0.01)	2 (0.01)	39 (<0.01)
Pancreas (C25)	667 (0.24)	238 (0.24)	85 (0.28)	154 (0.25)	423 (0.25)	382 (0.24)	31 (0.21)	990 (0.24)
Ill-defined digestive organs (C26)	128 (0.05)	47 (0.05)	23 (0.08)	35 (0.06)	81 (0.05)	78 (0.05)	4 (0.03)	198 (0.05)

Data are n (%).

Table S3. Cohort baseline characteristics categorized by daily sleep duration

Characteristic	Daily sleep duration			Total
	Normal	Short	Long	
Participants	278 199 (68·4)	98 381 (24·2)	30 004 (7·4)	406 584
Age	56·06 ± 8·15	56·12 ± 7·84	58·25 ± 8·08	56.23 ± 8·09
Townsend index	-1·58 ± 2·92	-1·01 ± 3·22	-1·18 ± 3·15	-1·41 ± 3·02
Fibre: daily intake				
Low	265 418 (95·4)	93 216 (94·8)	28 479 (94·9)	387 113 (95·2)
High	12 781 (4·6)	5 165 (5·2)	1 525 (5·1)	19 471 (4·8)
Fruits and vegetables: daily intake				
Low	117 041 (42·1)	42 209 (42·9)	12 802 (42·7)	172 052 (42·3)
High	161 158 (57·9)	56 172 (57·1)	17 202 (57·3)	234 532 (57·7)
Meat: weekly servings				
Low	193 513 (69·6)	67 721 (68·8)	19 855 (66·2)	281 089 (69·1)
High	84 686 (30·4)	30 660 (31·2)	10 149 (33·8)	125 495 (30·9)
Healthy diet score				
0	41 089 (14·8)	15 221 (15·5)	4 789 (16·0)	61 099 (15·0)
1	116 488 (41·9)	41 128 (41·8)	12 949 (43·2)	170 565 (42·0)
2	110 902 (39·9)	38 166 (38·8)	11 165 (37·2)	160 233 (39·4)
3	9 720 (3·5)	3 866 (3·9)	1 101 (3·7)	14 687 (3·6)
Race and Ethnicity				
White	267 314 (96·1)	91 625 (93·1)	28 664 (95·5)	387 603 (95·3)
Asian	5 489 (2·0)	2 560 (2·6)	649 (2·2)	8 698 (2·1)
Caribbean or African	3 023 (1·1)	2 829 (2·9)	399 (1·3)	6 251 (1·5)
Others	2 373 (0·9)	1 367 (1·4)	292 (1·0)	4 032 (1·0)
Educational level				
No qualification	39 044 (14·0)	18 654 (19·0)	7 516 (25·0)	65 214 (16·0)
Any other qualification	139 479 (50·1)	50 844 (51·7)	14 920 (49·7)	205 243 (50·5)
University degree	99 676 (35·8)	28 883 (29·4)	7 568 (25·2)	136 127 (33·5)
Region of the assessment centre				
England	246 636 (88·7)	87 575 (89·0)	26 391 (88·0)	360 602 (88·7)
Scotland	11 132 (4·0)	4 122 (4·2)	1 331 (4·4)	16 585 (4·1)
Wales	20 431 (7·3)	6 684 (6·8)	2 282 (7·6)	29 397 (7·2)
Alcohol consumption				
Not current	18 214 (6·5)	8 974 (9·1)	3 118 (10·4)	30 306 (7·5)
< 3 times per week	132 423 (47·6)	49 543 (50·4)	14 716 (49·0)	196 682 (48·4)
≥ 3 times per week	127 562 (45·9)	39 864 (40·5)	12 170 (40·6)	179 596 (44·2)
Pack-years [#]	21·57 ± 17·3	24·74 ± 19·73	26·66 ± 20·52	22·83 ± 18·34
Smoking status				
Never	157 669 (56·7)	52 413 (53·3)	15 499 (51·7)	225 581 (55·5)
Previous	95 214 (34·2)	34 004 (34·6)	11 035 (36·8)	140 253 (34·5)
Current	25 316 (9·1)	11 964 (12·2)	3 470 (11·6)	40 750 (10·0)
Type 2 diabetes	19 720 (7·1)	9 654 (9·8)	3 973 (13·2)	33 347 (8·2)
BMI status				
Normal weight	96 507 (34·7)	28 971 (29·4)	8 454 (28·2)	133 932 (32·9)
Underweight	1 346 (0·5)	538 (0·5)	154 (0·5)	2 038 (0·5)
Overweight	119 698 (43·0)	41 103 (41·8)	12 541 (41·8)	173 342 (42·6)
Obesity class I	44 779 (16·1)	19 239 (19·6)	6 057 (20·2)	70 075 (17·2)
Obesity class II	11 742 (4·2)	5 930 (6·0)	1 950 (6·5)	19 622 (4·8)
Obesity class III	4 127 (1·5)	2 600 (2·6)	848 (2·8)	7 575 (1·9)
Physical activity				
Low	41 303 (14.8)	16 277 (16.5)	5 313 (17.6)	62 893 (15.5)
Moderate	97 187 (34.9)	31 306 (31.8)	9 947 (33.2)	138 440 (34.0)
High	95 479 (34.4)	33 152 (33.8)	9 202 (30.7)	137 833 (33.9)
Missing	44 230 (15.9)	17 646 (17.9)	5 542 (18.5)	67 418 (16.6)

Data are mean ± SD or n (%). [#] Pack -years = Number of packs per day * Number of years smoking.

Table S4. Effects of eating habits on digestive organs cancer mortality.

Eating habit		Model A		Model B		Model B + Smoking		Model B + BMI status		Model B + T2D	
		**HR (95% CI)**	***P*-value**	**HR (95% CI)**	***P*-value**	**HR (95% CI)**	***P*-value**	**HR (95% CI)**	***P*-value**	**HR (95% CI)**	***P*-value**
Fibre											
	Low	Ref		Ref		Ref		Ref		Ref	
	High	0·92 (0·79-1·07)	0·304	0·88 (0·75-1·03)	0·105	0·89 (0·76-1·04)	0·138	0·88 (0·76-1·03)	0·116	0·88 (0·75-1·02)	0·098
Fruits and vegetables											
	Low	Ref		Ref		Ref		Ref		Ref	
	High	0·92 (0·87-0·98)	0·013	0·89 (0·84-0·95)	<0·001	0·93 (0·87-0·99)	0·022	0·89 (0·83-0·95	0·001	0·89 (0·83-0·95)	<0·001
Meat											
	Low	0·78 (0·73-0·83)	<0·001	0·90 (0·84-0·96)	0·001	0·93 (0·87-0·99)	0·032	0·92 (0·86-0·99)	0·019	0·92 (0·86-0·99)	0·018
	High	Ref		Ref		Ref		Ref		Ref	

Model A considered only the specific eating habit. Model B was adjusted for sleep duration, fibre daily intake, fruits and vegetables daily intake, meat weekly intake, sex, age, Townsend index, region of assessment centre, ethnicity, educational level, and alcohol consumption frequency.

Table S5. Multiplicative interactions with sex and age

Interaction term	P value
Sleep duration * Sex	0.166
Sleep duration * Age	0.913
Healthy diet score * Sex	0.775
Healthy diet score * Age	0.215
Fruits and vegetables * Sex	0.681
Fruits and vegetables * Age	0.138
Meat * Sex	0.548
Meat * Age	0.432
Fibre * Sex	0.447
Fibre * Age	0.222
Sleep duration * Healthy diet score * Sex	0.749
Sleep duration * Healthy diet score * Age	0.649
Sleep duration * Fruits and vegetables * Sex	0.286
Sleep duration * Fruits and vegetables * Age	0.290
Sleep duration * Meat * Sex	0.190
Sleep duration * Meat * Age	0.091
Sleep duration * Fibre * Sex	0.705
Sleep duration * Fibre * Age	0.782

Interaction terms were added to Model B, which was adjusted for sleep duration, healthy diet score (or fibre daily intake, fruits and vegetables daily intake, meat weekly intake), sex, age, Townsend index, region of assessment centre, ethnicity, educational level, and alcohol consumption frequency.

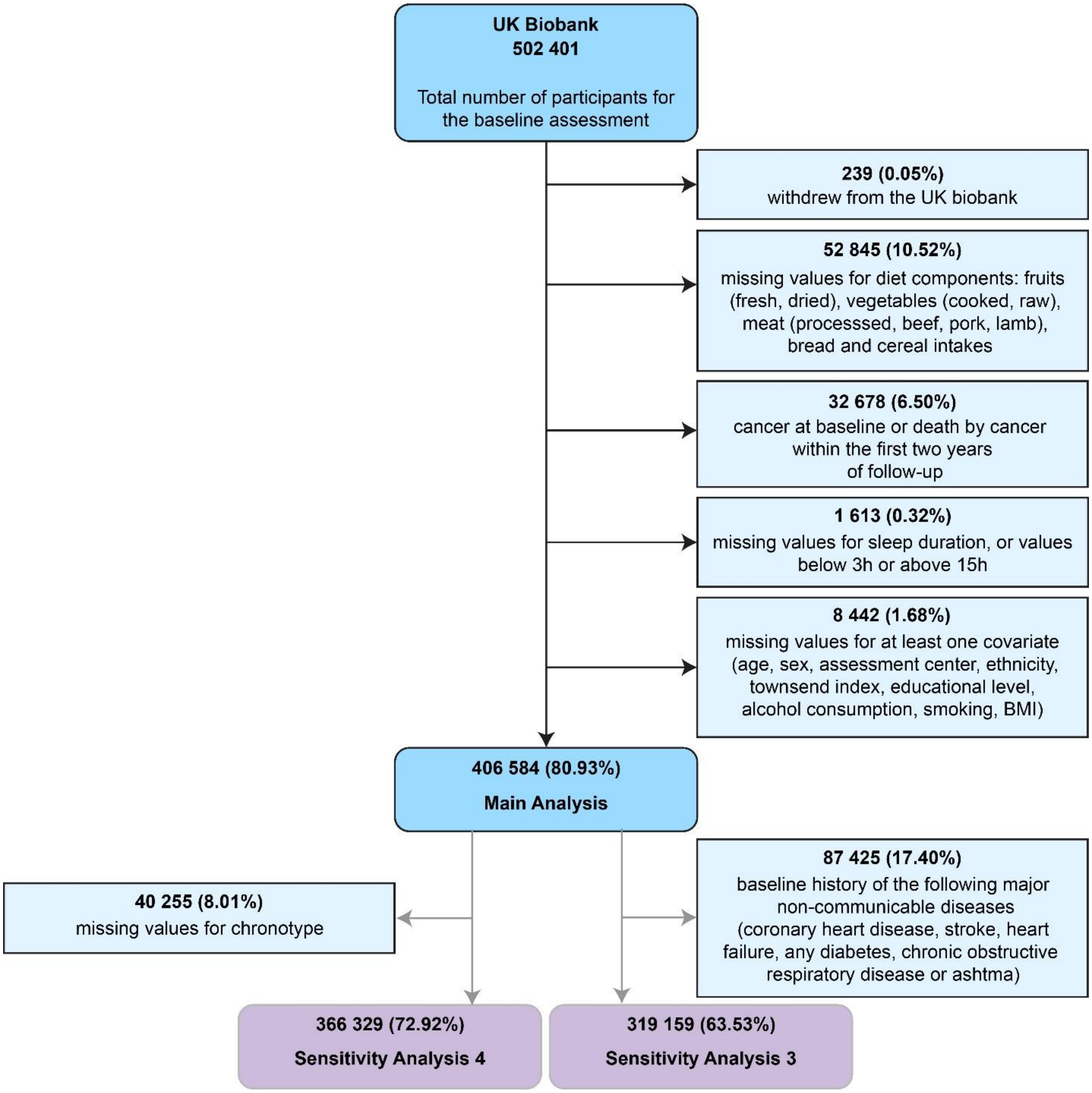

Figure S1. STROBE diagram.

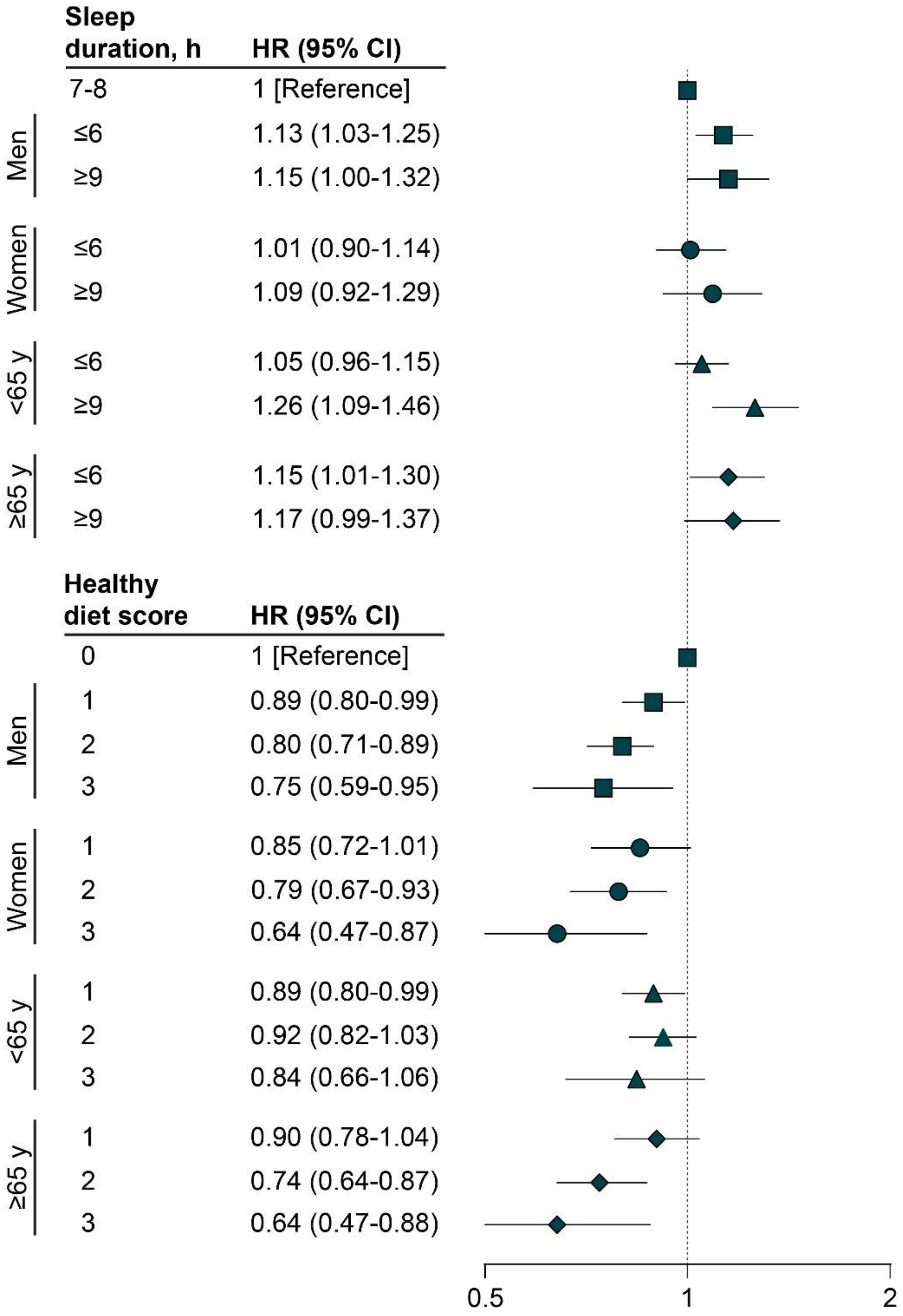

Figure S2. Association of daily sleep duration and healthy diet score with digestive system cancer mortality stratified by sex and age. Model B was adjusted for sleep duration, healthy diet score, sex OR age, Townsend index, region of assessment centre, ethnicity, educational level, and alcohol consumption frequency.
